# Lateral Fluid Percussion Injury Causes Sex-Specific Deficits in Anterograde but Not Retrograde Memory

**DOI:** 10.3389/fnbeh.2022.806598

**Published:** 2022-02-04

**Authors:** Julie Fitzgerald, Samuel Houle, Christopher Cotter, Zachary Zimomra, Kris M. Martens, Cole Vonder Haar, Olga N. Kokiko-Cochran

**Affiliations:** ^1^Department of Neuroscience, College of Medicine, The Ohio State University, Columbus, OH, United States; ^2^Institute for Behavioral Medicine Research, Neurological Institute, The Ohio State University, Columbus, OH, United States

**Keywords:** traumatic brain injury, anterograde memory, retrograde memory, behavior, memory

## Abstract

Cognitive impairment is a common symptom after traumatic brain injury (TBI). Memory, in particular, is often disrupted during chronic post-injury recovery. To understand the sex-specific effects of brain injury on retrograde and anterograde memory, we examined paired associate learning (PAL), spatial learning and memory, and fear memory after lateral fluid percussion TBI. We hypothesized that male and female mice would display unique memory deficits after TBI. PAL task acquisition was initiated via touchscreen operant conditioning 22 weeks before sham injury or TBI. Post-injury PAL testing occurred 7 weeks post-injury. Barnes maze and fear conditioning were completed at 14- and 15-weeks post-injury, respectively. Contrary to our expectations, behavioral outcomes were not primarily influenced by TBI. Instead, sex-specific differences were observed in all tasks which exposed task-specific trends in male TBI mice. Male mice took longer to complete the PAL task, but this was not affected by TBI and did not compromise the ability to make a correct choice. Latency to reach the goal box decreased across testing days in Barnes maze, but male TBI mice lagged in improvement compared to all other groups. Use of two learning indices revealed that male TBI mice were deficient in transferring information from 1 day to the next. Finally, acquisition and contextual retention of fear memory were similar between all groups. Cued retention of the tone-shock pairing was influenced by both injury and sex. Male sham mice displayed the strongest cued retention of fear memory, evidenced by increased freezing behavior across the test trial. In contrast, male TBI mice displayed reduced freezing behavior with repetitive tone exposure. An inverse relationship in freezing behavior to tone exposure was detected between female sham and TBI mice, although the difference was not as striking. Together, these studies show that retrograde memory is intact after lateral TBI. However, male mice are more vulnerable to post-injury anterograde memory deficits. These behaviors were not associated with gross pathological change near the site injury or in subcortical brain regions associated with memory formation. Future studies that incorporate pre- and post-injury behavioral analysis will be integral in defining sex-specific memory impairment after TBI.

## Introduction

Each year, more than 60 million individuals worldwide suffer traumatic brain injury (TBI) resulting in long-lasting effects (Dewan et al., 2018). Reports show that symptoms in 50% of persons with TBI stayed the same or worsened within the first 5 years after injury (CDC, 2021). Therefore, TBI is not an isolated event but rather part of a temporal process. Post-injury cognitive dysfunction is one of the most common symptoms in survivors. This can include impairments in memory, processing speed, and executive function (Rabinowitz and Levin, 2014), which encompasses behavioral flexibility, impulsivity, and decisiveness (see review, Ozga et al., 2018).

Experimental models of TBI are a valuable resource for defining post-injury cognitive change. For example, cognitive impairment is reported in rodents after weight drop (Beaumont et al., 1999, 2000), fluid percussion, controlled cortical impact (see review, Paterno et al., 2017), blast (Goldstein et al., 2012; Gerson et al., 2016), rotational acceleration (see review, McNamara et al., 2020) and repetitive (Nolan et al., 2018; Bachstetter et al., 2020; Sloley et al., 2021) brain injury. Cognition is commonly assessed through the measurement of spatial learning and memory in well-established behavioral tests, including Morris water maze (MWM), Barnes maze, radial arm maze (RAM), Y-maze, T-maze, and novel object recognition (NOR) (see review, Shultz et al., 2020). Most experimental TBI studies include post-injury behavioral testing. This results in examination of a cognitive deficit that is acquired after brain injury, preferentially identifying changes in anterograde memory. Additionally, most studies sum or average the primary behavioral dependent variable of interest over the entire testing trial. Together, these strategies result in a restricted, and often global, view of post-injury cognitive function.

Investigators have an opportunity to design more sophisticated behavioral studies around experimental TBI. Specifically, the controlled nature of experimental modeling allows investigators to manipulate both pre- and post-injury learning experiences. Quantitative analysis of within- and between-trial performance provides a way to define changes in learning and memory over time. Prioritizing these strategies aligns with the perspective of brain injury as a chronic health condition and shifts the focus of behavioral testing toward understanding time dependent changes in recovery. Consequently, one can begin to appreciate the different stages of learning and memory and even interpret encoding, storage, and retrieval of information. Combining detailed behavioral analysis with the wealth of data on specific brain structures and molecular pathways that mediate memory formation can further elucidate new therapeutic targets to improve chronic outcome.

The goal of the current study was to complete a comprehensive analysis of post-injury cognitive function. The study design included both pre- and post-injury behavioral analysis, which allowed us to evaluate retrograde and anterograde memory. Moreover, we included three cognitive tests to assess hippocampal-dependent memory. First, all mice were trained on a touchscreen operant conditioning paired associate learning (PAL) task 22 weeks before sham injury or TBI. The PAL touchscreen task models the Cambridge Neuropsychological Test Automated Battery (CANTAB) test in humans and evaluates the association of an object with a distinct spatial location (Bussey et al., 2008). PAL testing 6 weeks post-injury assessed memory of a previously learned task, i.e., retrograde memory of the object-location association. Second, Barnes maze testing began 13 weeks post-injury, 2 weeks after PAL testing finished. In this experimental design, post-injury Barnes maze testing assessed memory of a recently learned task, i.e., anterograde memory of the goal box location. Finally, fear conditioning was used to evaluate associative memory 14 weeks post-injury. We hypothesized that male and female mice would display unique memory deficits after TBI. Together, these studies aim to emphasize the value of pre- and post-injury behavioral analysis in defining time dependent changes in learning and memory after brain injury.

## Materials and Methods

### Experimental Study Design

**FIGURE 1:**
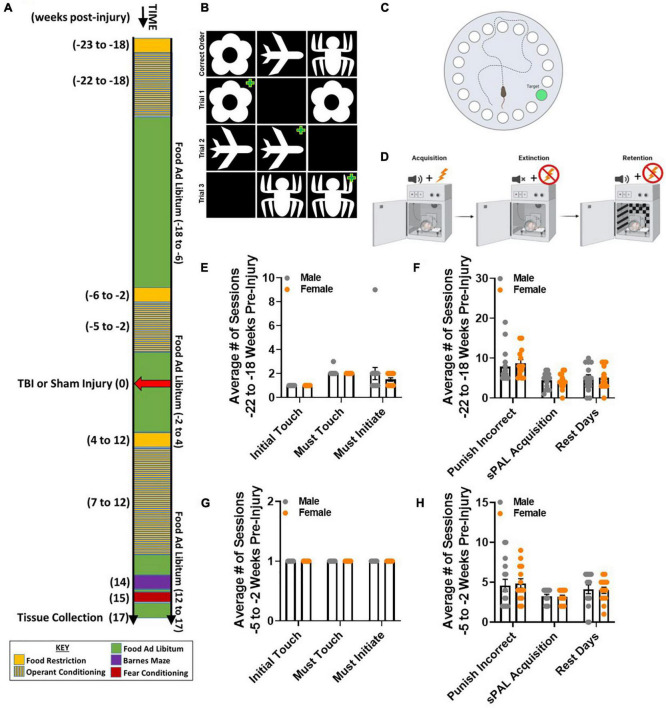
Acquisition of paired associate learning (PAL) is similar between naïve male and female mice. **(A)** Experimental timeline spanning approximately 40 weeks, including both pre- and post-injury behavioral testing to evaluate retrograde and anterograde memory, respectively. **(B)** Cartoon of the PAL task, displaying the correct order of images: flower, plane, and spider. Three example trials in which two of the same image are shown. To make a correct choice, a mouse must touch the image that is in the correct location. A green plus symbols identifies the correct choice. **(C)** Cartoon aerial view of the Barnes maze. Extra-maze cues are placed around the elevated maze to facilitate measurement of spatial reference memory across 5 testing days. Latency to locate the hole with a goal box is the primary dependent variable of interest. Shorter latency across testing days indicates improved performance. **(D)** Cartoon of conditioning equipment to measure fear memory. During acquisition, a mouse is exposed to multiple tone-shock pairings. During extinction, a mouse is exposed to the same testing environment as acquisition with no tone-shock pairings. During retention, a mouse is placed in a novel environment with the same tone-shock pairing as during the acquisition phase. Time freezing is the primary dependent variable of interest. **(E–H)** Male and female mice did not differ in number of sessions for Initial Touch, Must Touch, Must Initiate, Punish Incorrect, or PAL Acquisition -22 to -18 weeks pre-injury or -5 to -2 weeks pre-injury. Rest days were similar across sexes and at both time points. *N* = 7–8/group; error bars indicate SEM. Panels **(C,D)** made with BioRender.

The primary objective of this study was to define sex specific cognitive changes following TBI between 7 and 17 weeks post-injury. This resulted in a 2 (male, female) × 2 (sham, TBI) factorial design. To determine if TBI disrupted retrograde memory, equal numbers of male and female mice acquired a paired associate learning (PAL) touchscreen learning task 22 weeks before injury. Retention of 80% accuracy in the paired associate touchscreen learning task was confirmed 5 weeks before injury. All mice received surgical preparation, and half of the mice in each sex received either a sham injury or a lateral fluid percussion TBI. Retrograde memory of the paired associated task was assessed 7–12 weeks post-injury. In order to determine if TBI disrupted anterograde memory, all mice learned two new cognitive tasks. Barnes maze was completed 14 weeks post-injury, and fear conditioning was completed 15 weeks post-injury. Brain tissue was collected from all mice 17 weeks post-injury (Figure 1A).

### Subjects

Equal numbers of 10-week-old male and female C57BL/6 mice were purchased from Charles River Laboratories (Wilmington, MA, United States). Mice were individually housed in The Ohio State University’s vivarium and maintained at 21°C under a 12-h light/12-h dark cycle. Following a 1-week habituation to the vivarium, all mice were food restricted during touchscreen operant conditioning training and testing. Otherwise, all mice had free access to food and water. Males and females were counterbalanced in all procedures and analyses. Two cohorts of *N* = 16 mice (8 males and 8 females) acquired the operant conditioning task, resulting in 32 mice total. One male TBI, one female sham, and one female TBI mouse had to be removed from the study after injury because of exclusion criteria (e.g., injured brain during surgery, compromised injury severity). Finally, *N* = 8 male sham, and *N* = 7 male TBI, female sham, and female TBI were used for all behavioral analyses. A random subset of mice from each group were used for histological analysis. All experiments were completed in accordance with principles set forth by the National Institutes of Health Guidelines for the Care and Use of Laboratory Animals and were approved by the Institutional Animal Care and Use Committee of The Ohio State University.

### Surgical Preparation and Lateral Fluid Percussion Injury

Surgical preparations followed as previously described (Kokiko-Cochran et al., 2016, 2017; Tapp et al., 2020). Briefly, mice were anesthetized with 4% isoflurane gas for 4 min. Once anesthetized, mice were shaved and fixed in a stereotaxic frame. Following sterilization of the skin, an incision was made at the midline of their skull. Sterile cotton swabs were used to clear fascia from the skull. Then, a drill was used to create a small guide notch on the right parietal bone approximately 4 mm to the right of midline, directly between lambda and bregma. This notch served as a guide for a trephine drill which was used to perform a 3 mm craniectomy over the parietal lobe while leaving the dura intact. Once the skull flap was removed, a modified leur-lock syringe, modified to be about 1 cm tall with an internal diameter of 3 mm, was affixed to the craniectomy using super glue and sealed using dental cement. The syringe hub was then filled with saline and mice were injected with 2 ml of saline subcutaneously before being removed from the stereotaxic frame and returned to their home cage.

The following day, mice were again anesthetized using 4% isoflurane for 4 min. At the end of the 4 min, the hub was filled with saline and mice were subsequently attached to a fluid percussion injury (FPI) device (Custom Design & Fabrication, Richmond, VA, United States). To induce a moderate injury, a weighted pendulum was allowed to swing from a pre-determined height and contact the end of the FPI once, creating a fluid pulse at a magnitude of 1.2 atm (Witcher et al., 2018, 2020) that was delivered down the length of the device and onto the dura. Hubs were removed after injury, and the incision was closed by staples. Half of the mice were randomly selected to receive this injury. For the sham population, mice were anesthetized and affixed to the FPI but did not receive a fluid pulse. They then immediately had their hubs removed and incisions closed. All mice had their injury severity assessed by measuring their latency to self-righting reflex following TBI or sham injury.

### Behavioral Testing

#### Touchscreen Operant Conditioning

The Bussey-Saksida Touchscreen Chamber (Campden Instruments) was used for the PAL task. This equipment consists of a trapezoidal-shaped chamber with perforated floors and a removable tray underneath. The longer side houses the touchscreen while the reward tray is along the shorter side. All training and testing stages are loaded through the ABET II software and data is saved automatically. Prior to training, mice were weighed for 3 consecutive days to calculate their mean free feeding weight. After weighing on the third day, food restriction started. Mice were provided a daily single food pellet between 2.8 and 3.2 g and weighed daily to ensure their weight was maintained between 85 and 90% of their mean free feeding weight. The mice were then introduced to the reinforcer (Strawberry Ensure) for 2 consecutive days. All training stages followed the manual provided by Campden Instruments with a few minor changes, Table 1. Training was conducted 5 days per week using the 3 × 1 black insert required for the PAL task.

**TABLE 1:** Touchscreen operant conditioning training stages.

Stage	Max # of trials	Max session length	Reward	Inter-trial interval	Days to reach criteria	Description of training	Notes	Pre-injury	Post-injury
Habituation 1	N/A	10 min	No	N/A	1	Acclimates mouse to chamber		x	
Habituation 2a	N/A	20 min	Yes	N/A	1	Acclimates mouse to chamber; Teaches mouse to associate reward with tray light and tone		x	
Habituation 2b	N/A	40 min	Yes	N/A	1	Acclimates mouse to chamber; Teaches mouse to associate reward with tray light and tone		x	
Initial touch	36	60 min	Yes	10 s	1	Mouse must touch screen to get reward (blank window: normal amount; correct window: 3×)		x	
Must touch	36	60 min	Yes	10 s	1–2	Mouse must touch white square to get reward (no response if blank window touched)		x	x
Must initiate	36	60 min	Yes	10 s	1–2	Mouse must nose poke and exit reward tray to initiate display of stimulus		x	
Punish incorrect	36	40 min	Yes	10 s	2–15	Teaches mouse there are correct and incorrect answers (house light on for incorrect)	27/36 (75%) of trials correct	x	x
PAL training	36	60 min	Yes	10 s	1	Mouse must determine correct location of novel stimuli/object to receive reward		x	
Rest						Done to avoid over-training		x	x
PAL full task	36	60 min	Yes	10 s	6 weeks	Mouse must determine correct location of novel stimuli/object to receive reward			x

##### Habituation

The Habituation stages occurred over 3 consecutive days. For Habituation 1, mice were placed in the chamber to acclimate for 10 min where no tone, light, or reinforcer were presented. For Habituation 2a and 2b, tone (software’s default setting, 3 KHz) and light were paired with reinforcer retrieval (software’s default setting, 7 μl for 280 ms). These sessions lasted 20 and 40 min, respectively. The reward tray light was switched on, and a tone played to indicate the reinforcer was dispensed. Upon retrieval of the reinforcer, a 10 s delay occurred before the light, tone, and reinforcer were presented again. This was repeated until the sessions ended.

##### Initial Touch Training

During this training stage a stimulus (white square) was paired with the reinforcer. The stimulus was shown randomly in 1 of the 3 windows. Nosepokes to the stimulus within 30 s were reinforced three times. Failure to nosepoke the white square resulted in removal of the stimulus followed by administration of 1 reinforcer. Collection of the reinforcer resulted in a 10 s inter-trial interval (ITI) followed by stimulus presentation. Training ended after 60 min or after 36 trials were completed.

##### Must Touch Training

During this training stage a stimulus (image of flower, plane, or spider) was shown randomly in 1 of the 3 windows. Nosepokes to the stimulus were reinforced followed automatically by a 10 s ITI. Nosepokes to the blank windows were not reinforced. This was repeated until 60 min was reached or after 36 trials were completed.

##### Must Initiate Training

This training stage continued as described above in Must Touch Training, but before the stimulus was presented the reward tray light was activated and a small amount of reinforcer was dispensed. Exit of the reward tray initiated each trial. This was repeated until 60 min was reached or after 36 trials were completed.

##### Punish Incorrect Training

During this training stage, the stimulus (image of flower, plane, or spider) was presented randomly in 1 of the 3 windows. Nosepokes to the correct window were reinforced followed by a 10 s ITI. Nosepokes to a blank window resulted in a 5 s time out where the stimulus was removed from the screen and the house light was switched on. Incorrect responses were not reinforced. After an incorrect response a correction trial followed where the stimulus was shown in the same location as the previous trial. This was repeated until the mouse made a correct response at which point the reinforcer was administered. This session ended after 40 min was reached or when 27 out of 36 trials were correct over 2 consecutive days. Correction trials were not included in the final trial count.

##### Paired Associate Learning Acquisition Training

During this training stage the same images of flower, plane, and spider were used, each with a correct location in 1 of the windows: flower, left; plane, middle; spider, right. After the trial was initiated, 2 of the same images were presented with 1 in the correct location and the other in the incorrect location (Figure 1B). Nosepokes to the image in the correct location were reinforced followed by a 10 s ITI. Nosepokes to the incorrect location or blank window were not reinforced. The session ended after 60 min or after 36 trials were completed. Following completion of this stage, some mice were rested to avoid overtraining and to make sure all were ready for surgery and injury at the same time. This meant those particular mice were only trained 2–3 days per week and rested the remaining days. All training stages were completed to criteria before the 3-month rest period. Previous studies reported that C56BL/6 mice failed to perform above chance levels in the PAL paradigm (Nichols et al., 2017). Therefore, retention of the task was confirmed 5 weeks before TBI or sham injury. All mice retained the task above chance levels and returned to free feeding before injury. Mice recovered with food and water *ad libitum* for 4 weeks post-injury before being food restricted for PAL testing. After 1 week of food restriction, all mice were re-trained on Must Touch and Punish Incorrect 5–6 weeks post-injury before moving to the full task 7 weeks post-injury.

##### Paired Associate Learning Full Task

This stage was a combination of PAL Acquisition and Punish Incorrect Trainings. Two of the same images were presented (flower, plane, and spider) in their correct and incorrect locations. A correct choice resulted in reinforcement; an incorrect choice resulted in the 5 s time out period. Correction trials followed all incorrect trials and were not included in the percent correct data. The session ended after 60 min or after 36 trials were completed. This was repeated 5 days per week for 6 weeks.

### Barnes Maze

Barnes maze is a memory task that requires mice to use spatial cues around an elevated platform to locate a hidden goal box. Barnes maze was performed 2 weeks following the completion of operant conditioning testing at 14 weeks post-injury. Cages were brought into the behavior suite 20–30 min prior to starting. The animals were exposed to bright light (clamp lamp positioned above the maze, 75W) throughout testing as well as white noise (66 dB) while on the maze. To help increase the motivation to enter the escape box, bedding from each cage was placed inside. The escape box was cleaned and replaced with the next cage’s bedding in between. Each mouse was placed in the middle of the maze at the start of each trial and allowed to explore for 2 min. Each trial ended when the mouse entered the escape box or after 2 min had elapsed. Immediately after the mouse entered the box, it was allowed to stay there for about 30 s. If the mouse did not reach the goal within 2 min, the experimenter gently guided the mouse to the escape box and left the mouse inside for about 30 s (Figure 1C). Once the mouse was placed back in its home cage, the maze and escape box were cleaned with 70% ethanol followed by testing of the next mouse. This was repeated until each animal had received 3 trials per day over 5 consecutive days. During these training days, the latency to goal, distance traveled, and time in goal perimeter were recorded by the ANY-maze tracking system (Stoelting). On day 6, the probe trial was conducted. During the probe trial, the escape box was removed, and the mouse was placed in the middle of the maze and allowed to explore for a fixed interval of 60 s. Latency to goal perimeter was the primary dependent variable of interest for the probe trial. Within day learning was quantified with the acquisition index, where *t*_F_ and *t*_L_ are the latency to reach the goal from the first and last trials of the day, respectively, and k is the number of days of training in the Barnes maze: ([*t*_F_*d*_1_ − *t*_L_*d*_1_] + … + [*t*_F_*d*_k_ − *t*_L_*d*_k_])/_k_. Between day learning was quantified with the savings index, where the difference is calculated between the latency to reach the goal on the last trial of a day and the first trial of the subsequent day: ([*t*_L_*d*_1_ − *t*_F_*d*_2_] + … + [*t*_L_*d*_k–1_ − *t*_F_*d*_k_])/_k_. Both learning indices have been described in previous publications (Kokiko-Cochran et al., 2016; Whiting and Kokiko-Cochran, 2016).

### Fear Conditioning

During fear conditioning, a conditional stimulus (CS; tone) is paired with an unconditional stimulus (US; footshock). With repeated CS-US pairings, mice display a conditional response (CR; freezing) to both the CS and training context. Fear conditioning was performed the week following Barnes maze at 15 weeks post-injury. The chamber was cleaned with 70% ethanol between mice. Total freezing time and freezing episodes were recorded daily via the ANY-maze tracking system. Default settings were used, including a minimum freeze duration of 1,000 ms, a “freezing on” threshold of 30, and a “freezing off” threshold of 40. Tone was set at 2 KHz, 80% volume. The protocol used consisted of 3 testing sessions over 3 days (Figure 1D).

#### Session 1, Acquisition

Following a 120 s acclimation, the animals were presented with a 2 kHz tone for 20 s. A 0.6 mA shock was administered during the last second of the tone. This 20 s tone and co-terminating shock were administered 5 times with a 30 s inter-trial interval. The session ended with a 60 s observation period.

#### Session 2, Context

Approximately 24 h after Session 1, the animals were placed in the chamber and activity was recorded for 180 s. During this time, no tone or shock was presented.

#### Session 3, Retention

This session took place approximately 24 h after Session 2 and was designed exactly as in Session 1, except no shock was administered. Additionally, the chamber was changed so that the environment was less recognizable to the animal. Changes included a vanilla extract scent, a solid white floor panel and black and white checkered wall inserts.

### Immunohistochemistry and Imaging Analysis

At 17 weeks post-injury, mice were euthanized by carbon dioxide asphyxiation and then transcardially perfused with ice-cold 1× PBS (pH 7.4). Brains were dissected from the skull and placed in 4% PFA for 72 h. Next, brains were placed in a 30% sucrose solution for 72 h and stored at 4°C. Brains were oriented and embedded in Optimal Cutting Temperature compound and sectioned at 30 μm using a Leica Biosystems cryostat. The brain sections were placed directly into wells with 1× PBS for 24 h. Sections were then transferred to cell plates containing cryoprotectant and stored at −20°C until antibody labeling.

For immunofluorescent labeling, sections were rinsed in 0.1% Triton X-100 in PBS (PBST) 3× for 10 min with rotation. Tissue was then blocked (5% normal donkey serum, 0.3% Triton X-100 in PBS) for 1 h at room temperature with constant rotation. Subsequently, sections were incubated overnight at 4°C in primary antibody with constant rotation: rabbit anti-mouse Iba1 (1:500; Wako Chemicals 019-19741) and mouse anti-mouse NeuN (1:2000; Abcam ab104224). Next, tissue was rinsed 3× in 1× PBST and incubated in corresponding fluorochrome-conjugated secondary antibody for 1 h (1:1000; Alexa Fluor 594 Donkey anti-mouse and 647 donkey anti-rabbit). Sections were then mounted and cover-slipped using Fluoromount-G (Invitrogen).

Iba1 and NeuN images were taken using a Leica SP8 confocal microscope (Leica Microsystems). Z stacked images with a step size of 2 μm were taken over the dentate gyrus of the hippocampus, the primary somatosensory cortex (referred to as lateral cortex), the dorsolateral thalamus, and basolateral amygdala ipsilateral to injury. We have shown that post-injury neuroinflammation persists in these brain regions following lateral FPI (Kokiko-Cochran et al., 2016, 2017). Additionally, several studies show that they are involved in associative and spatial memory (Van Groen et al., 2002; Clark and Harvey, 2016; Guo et al., 2017; Hainmueller and Bartos, 2020). 2–3 images were acquired per region, per animal at 20× magnification. Both Iba1 and NeuN image percent-area were quantified by blinded researchers using a fully open-source version of the NIH’s Image J 2.3.051 (FIJI). Differences in threshold values between Iba1 images were controlled by using an unbiased thresholding algorithm named “Moments” (Tsai, 1985). This auto-thresholding algorithm has been incorporated into FIJI and was verified to be accurate for Iba1 images against hand scores by experienced researchers. Values were averaged for each region within each animal to normalize against any outliers.

### Statistical Analysis

All statistical analysis was completed in GraphPad Prism 9.0. Two-way ANOVA with Tukey correction for multiple comparisons was performed with injury (sham and TBI) and sex (male and female) as independent variables. Main effects of injury and sex as well as interaction effects were considered. Three-way ANOVA with Tukey correction for multiple comparisons was performed with injury (sham and TBI), sex (male and female) and time (testing day/week or testing trial) as independent variables. Main effects of injury, sex, time, and interaction effects were considered. Statistical significance was determined as *p* < 0.05. All data are presented as mean ± standard error of the mean (SEM).

## Results

### Acquisition of Paired Associate Learning Is Similar Between Naïve Male and Female Mice

Initial training on the PAL task began 2 weeks after the mice arrived at The Ohio State University and 1 week after food restriction began. The total training time was 4 weeks. Following habituation to the touchscreen operant chamber, all mice completed 5 training stages: Initial Touch, Must Touch, Must Initiate, Punish Incorrect, and PAL acquisition. Rest sessions were introduced to avoid over training. No statistically significant differences were observed between male and female mice during initial acquisition of the PAL procedures 22–18 weeks before injury, which corresponds to about 5 months before sham injury or TBI (Figures 1E,F). Similar performance on the PAL task was observed between male and female mice 5 weeks before sham or TBI (Figures 1G,H). These data show that naïve male and female mice acquire the PAL task similarly with retention for several months.

### Traumatic Brain Injury Induces a Longer Righting Time Than Sham Injury

**FIGURE 2:**
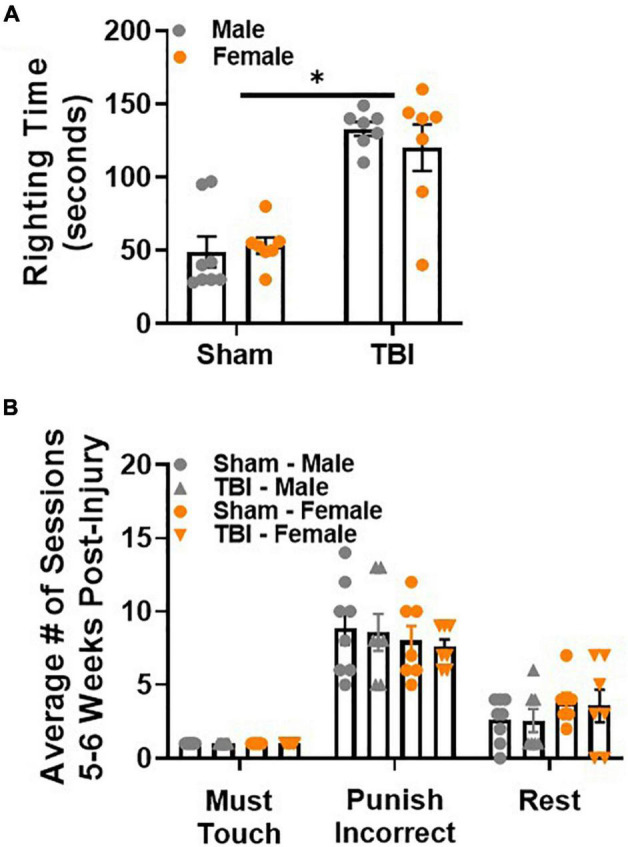
Paired associate learning procedural memory is intact after sham injury or TBI. **(A)** TBI induces a longer righting time than sham injury. **(B)** Sham and TBI mice, regardless of sex, perform similarly on procedural PAL tasks 5 weeks post-injury. *N* = 7–8/group; error bars indicate SEM. Main effects are identified in the figure, unless an interaction effect was identified. *p < 0.05.

Righting time to regain consciousness was longer after TBI than sham injury, *F*(1,25) = 54.98, *p* < 0.01, Figure 2A. This response was not influenced by sex.

### Paired Associate Learning Procedural Memory Is Intact After Sham Injury or Traumatic Brain Injury

To confirm that all mice retained procedural memory of the PAL task, 2 training stages were repeated: must touch, punish incorrect. Rest sessions were introduced to avoid overtraining. Male and female mice performed similarly in both training stages regardless of injury group (Figure 2B). Rest sessions were also similar between both sexes regardless of injury group. Together, these data demonstrate that procedural memory of the PAL task is intact after sham injury or TBI.

### Sex-Specific Response Time Does Not Significantly Compromise Retrograde Memory After Traumatic Brain Injury

**FIGURE 3:**
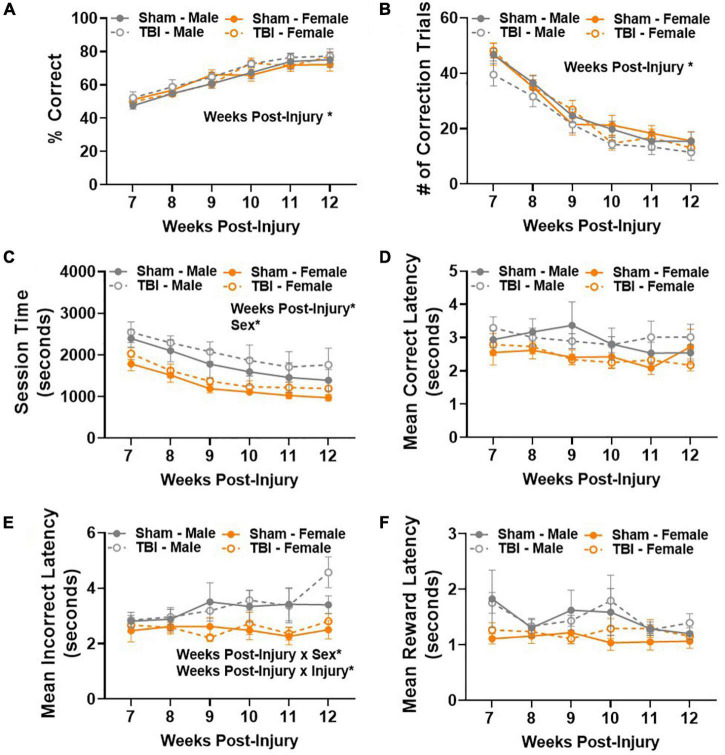
Sex-specific response time does not significantly compromise retrograde memory after TBI. **(A)** Percent correct increased in all experimental groups 7–12 weeks post-injury. **(B)** Number of correction trials decreased in all experimental groups 7–12 weeks post-injury. **(C)** Average session time decreased in all experimental groups 7–12 weeks post-injury, but males took longer than females regardless of injury group. **(D)** Mean correct latency did not change between experimental groups 7–12 weeks post-injury. **(E)** Sex- and injury-dependent changes emerged in mean incorrect latency 7–12 weeks post-injury. **(F)** Mean collection latency did not differ between experimental groups 7–12 weeks post-injury. *N* = 7–8/group; error bars indicate SEM. Main effects are identified in the figure, unless an interaction effect was identified. *p < 0.05.

Retrograde memory was assessed in a touchscreen operant conditioning PAL task beginning 7 weeks post-injury. There was a statistically significant improvement in performance between 7–12 weeks post-injury [main effect of weeks post-injury, *F*(5,125) = 67.41, *p* < 0.01; Figure 3A]. The percentage of correct choices increased from approximately 50–80% in all experimental groups over 6 weeks, meeting the criterion for pre-injury performance and indicating that retrograde memory of a previously learned task was intact. We considered a variety of other dependent variables to confirm integration of the learned task from day to day. The number of correction trials significantly decreased in all experimental groups between testing weeks, *F*(5,125) = 143.5, *p* < 0.01 (Figure 3B), confirming that mice became more efficient at making correct choices over time.

Next, we examined average session time. A session ended after a maximum of 36 trials or 60 min passed. Main effects of weeks post-injury, *F*(5,125) = 23.23, *p* < 0.01, and sex, *F*(1,25) = 7.52, *p* < 0.05, were identified (Figure 3C). Together, these data show that efficiency in making correct choices resulted in shorter session times with repeated testing. However, male mice took longer to complete a session than female mice. We also examined the average latency to make a correct or incorrect choice in the PAL task. No statistically significant differences were identified in the amount of time it took to make a correct choice (Figure 3D). A main effect of weeks post-injury, *F*(5,125) = 2.73, *p* < 0.05, and sex, *F*(1,25) = 6.38, *p* < 0.05, were identified in average latency to make an incorrect choice (Figure 3E). In addition, weeks post-injury × sex, *F*(5,125) = 2.57, *p* < 0.05, and weeks post-injury × injury, *F*(5,125) = 2.55, *p* < 0.05, interaction effects were also identified (Figure 3E). These results are largely attributed to male mice taking longer to make an incorrect choice. In addition, the latency to make an incorrect choice increased over testing weeks in TBI mice. *Post hoc* comparisons show that male TBI mice are the only experimental group to take significantly more time to make an incorrect choice between weeks 7 and 11, *p* < 0.05. At 11 weeks post-injury, male TBI mice have a significantly longer mean incorrect latency than female sham mice, *p* < 0.05. Finally, no significant differences were identified in mean collection latency, i.e., the amount of time spent retrieving a reinforcer after a correct choice was made (Figure 3F). Together, these data show that male mice took longer to complete the post-injury PAL task, but this did not compromise their ability to make correct choices.

### Male Traumatic Brain Injury Mice Display Unique Spatial Memory Deficits 14 Weeks Post-injury Characterized by Impaired Transfer of Information Between Testing Days

**FIGURE 4:**
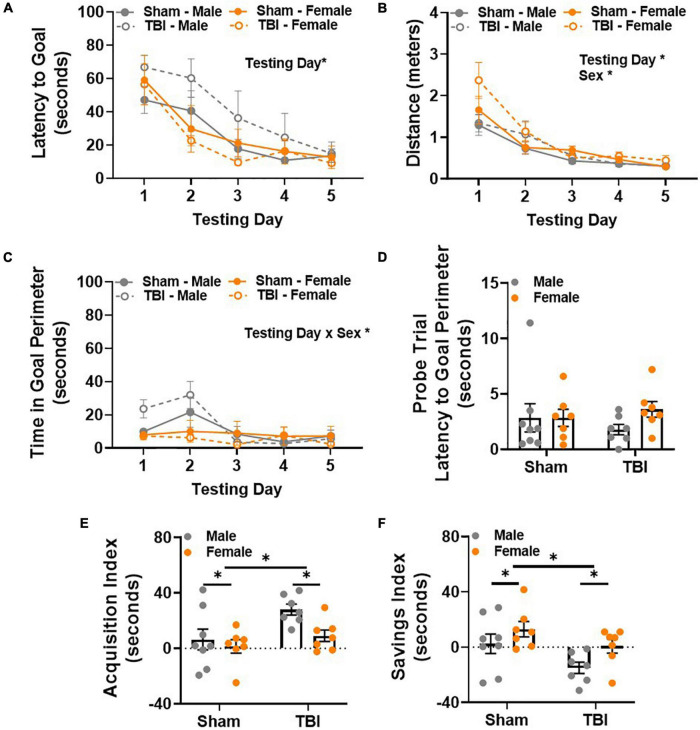
Male TBI mice display unique spatial memory deficits 14 weeks post-injury characterized by impaired transfer of information between testing days. **(A)** Latency to reach the Barnes maze goal box decreases over testing days. **(B)** Distance to reach the Barnes maze goal box decreases over testing days. **(C)** Male TBI mice spend more time in the goal perimeter during the first 2 days of testing, but this resolves over time. **(D)** Latency to the goal perimeter did not differ between experimental groups during the probe trial. **(E)** Average acquisition index shows that latency to reach the goal box significantly decreases in male TBI mice compared to all other groups. **(F)** Average savings index shows that latency to reach the goal box significantly increases in male TBI mice compared to all other groups. *N* = 7–8/group; error bars indicate SEM. Main effects are identified in the figure, unless an interaction effect was identified. *p < 0.05.

Spatial reference memory was evaluated in the Barnes maze 14 weeks post-injury. Latency to reach the goal box was considered the primary dependent variable of interest. All experimental groups learned the task, as indicated by decreased latency to reach the goal box across days [main effect of testing day, *F*(1,100) = 26.49, *p* < 0.01, Figure 4A]. As expected, average distance decreased across testing days in all experimental groups [main effect of testing day, *F*(4,100) = 37.81, *p* < 0.01, Figure 4B]. However, a main effect of sex, *F*(1,25) = 4.84, *p* < 0.05, was also detected in distance traveled (Figure 4B). This was likely related to performance on testing day 1, where female TBI mice traveled the most (*M* = 2.37, *SEM* = 0.21) and male sham mice traveled the least (*M* = 1.29, *SEM* = 0.25). Differences on the first two testing days were also apparent when considering time spent in the goal perimeter. Goal perimeter was operationally defined at the 1.5 inch perimeter around the goal hole on top of the Barnes maze. Analyzing time spent in the goal perimeter provided a way to identify experimental groups that traverse to the goal but do not actually enter the goal box or sit near the goal box for a period of time before entering. Here, a main effect of testing day, *F*(4,100) = 7.68, *p* < 0.01, and a testing day × sex interaction effect, *F*(4,100) = 5.21, *p* < 0.01, were detected (Figure 4C). These results were largely attributed to male mice spending the most time in the goal perimeter on testing days 1 and 2. *Post hoc* comparisons showed that male TBI mice (*M* = 31.96, *SEM* = 8.15) spent more time in the goal perimeter than female TBI mice (*M* = 6.33, *SEM* = 1.77) on testing day 2. The amount of time spent in the goal perimeter decreased in male mice after testing day 2. *Post hoc* analysis showed that the day 2 perimeter time for male TBI mice was significantly longer than the perimeter time for all experimental groups on testing days 3–5. Together, these data suggest that male TBI mice explore or sit within the goal perimeter more than other experimental groups. This behavior may contribute to longer latencies to complete the task (i.e., enter the goal box) during the first 2 days of testing but resolves thereafter.

All experimental groups performed similarly on the fifth day of Barnes maze testing, i.e., no differences in latency or distance traveled were observed. As expected, no differences in latency to the goal perimeter were detected in the probe trial on testing day 6 (Figure 4D). All but one mouse reached the goal perimeter within 10 s of starting the probe trial. Together, these data suggest that exploration time in the goal perimeter decreases in male TBI mice with repeated testing. This observation does not explain why latency to the goal would remain longer in male TBI mice on testing days 3 and 4 though.

For this reason, two learning indices were used to evaluate within and between day learning in the Barnes maze. The acquisition index measures within day learning and positive values represent improvement in performance between trial 1 and trial 3 within any given testing day. Main effects of injury, *F*(1,25) = 7.00, *p* < 0.05, and sex, *F*(1,25) = 4.65, *p* < 0.05, were observed (Figure 4E). Overall, the acquisition index was higher in TBI mice compared to shams, and lower in female mice compared to male mice. Next, we used the savings index to measure between day learning. Here, positive values represent improvement between the previous day’s trial 3 and trial 1 of the new day to provide an indirect measurement of memory consolidation between testing days. Main effects of injury, *F*(1,25) = 6.85, *p* < 0.05, and sex, *F*(1,25) = 5.42, *p* < 0.05, were observed (Figure 4F). Overall, the savings index was lower in TBI mice compared to shams, and higher in female mice compared to male mice. Together, these data demonstrate that male and female mice maintained unique within and between day learning profiles 14 weeks post-injury. Male mice showed significant improvement within testing days, but this was not sufficient to overcome deficits in transferring information from 1 day to the next. For male TBI mice, this may have contributed to generally longer latencies to reach the Barnes maze goal.

### Traumatic Brain Injury Results in Sex-Specific Retention Deficits of Fear Memory 15 Weeks Post-injury

**FIGURE 5:**
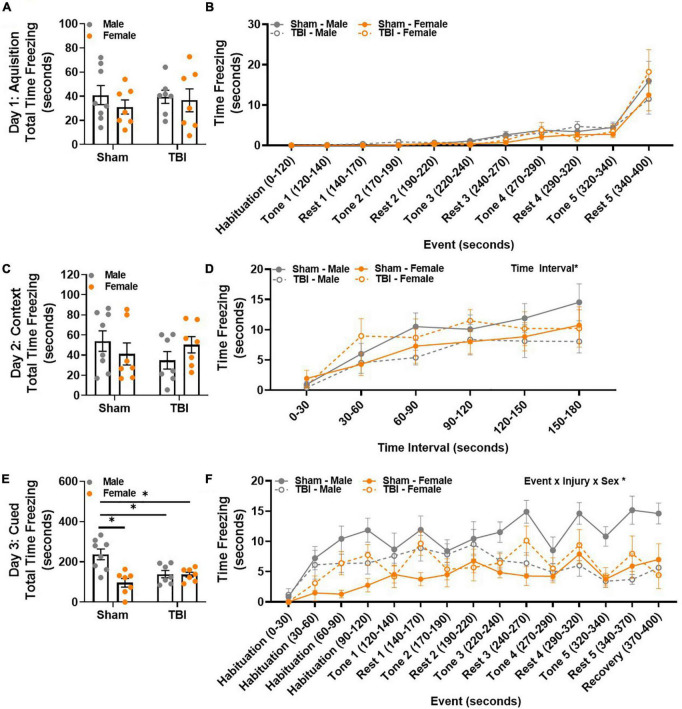
TBI results in sex-specific retention deficits of fear memory 15 weeks post-injury. **(A)** Total time freezing was similar between all groups during the acquisition phase. **(B)** Freezing in response to tone-rest events was similar between all groups during the acquisition phase. **(C)** Total time freezing was similar between all groups during the contextual/extinction phase. **(D)** Freezing increased in all experimental groups over time. **(E)** Male sham mice spent the most total time freezing during the cued/retention phase. **(F)** Freezing in response to tone-rest events was uniquely influenced by both injury and sex, with male sham mice freezing the most over time. *N* = 7–8/group; error bars indicate SEM. Main effects are identified in the figure, unless an interaction effect was identified. *p < 0.05.

Contextual and cued fear conditioning were used to assess fear memory. The 3-day behavioral paradigm included an acquisition day in which mice were exposed to an auditory cue (tone) that co-terminated with an electric footshock 5 times. The following day, freezing behavior in response to the same testing environment was used to measure contextual fear memory. Finally, on the third testing day, mice were placed in a novel environment and exposed to the same auditory cue from the acquisition phase, but no footshock was delivered. Freezing behavior in response to the auditory cue was used to measure cued fear memory. Total time freezing was similar between all experimental groups during the acquisition phase on day 1 (Figure 5A). To identify potential changes in behavior across the acquisition phase, time freezing was examined in response to trial events, i.e., repeated tone/footshock – rest pairings. No significant differences in time freezing were identified between experimental groups when trial event was considered (Figure 5B). Therefore, learning was similar between experimental groups.

Total time freezing was similar between all experimental groups during the contextual memory assessment (Figure 5C). A main effect of time interval, *F*(5,125) = 17.09, *p* < 0.01, emerged when time freezing was evaluated in 30-s time bins (Figure 5D). All experimental groups displayed an increase in freezing over time. A main effect of sex was observed in total time freezing during the cued memory assessment, *F*(1,25) = 11.67, *p* < 0.01 (Figure 5E). An interaction effect between injury × sex was also identified, *F*(1,25) = 10.83, *p* < 0.01 (Figure 5E). *Post hoc* comparisons confirmed that this was because male sham mice spent more time freezing than any other group (male sham, *M* = 237.04, *SEM* = 26.90; male TBI, *M* = 138.70, *SEM* = 18.15; female sham, *M* = 96.14, *SEM* = 21.45; female TBI, *M* = 136.07, *SEM* = 11.99). Strikingly, time freezing in response to trial events, i.e., repeated tone – rest pairings, was largely influenced by event, event × injury × sex interaction effect, *F*(14,350) = 2.74, *p* < 0.01 (Figure 5F). *Post hoc* comparisons show that male sham mice are the only experimental group to increase freezing behavior between habituation 0–30 s and recovery 370–400 s. In addition, male sham mice increase their freezing behavior in response to tone 4. While there is some fluctuation in freezing behavior thereafter, male sham mice continue to freeze more than any other experimental group throughout the remainder of the testing trial (*p* < 0.05). Therefore, male sham and male TBI mice display divergent freezing behavior in response to repeated tone-shock pairings. This same response is not observed in female sham and female TBI mice. Together, these data suggest that sex-specific injury freezing behavior changes over time.

### Gross Pathological Change Is Absent Near the Site of Injury and in the Basolateral Amygdala 17 Weeks Post-traumatic Brain Injury

**FIGURE 6:**
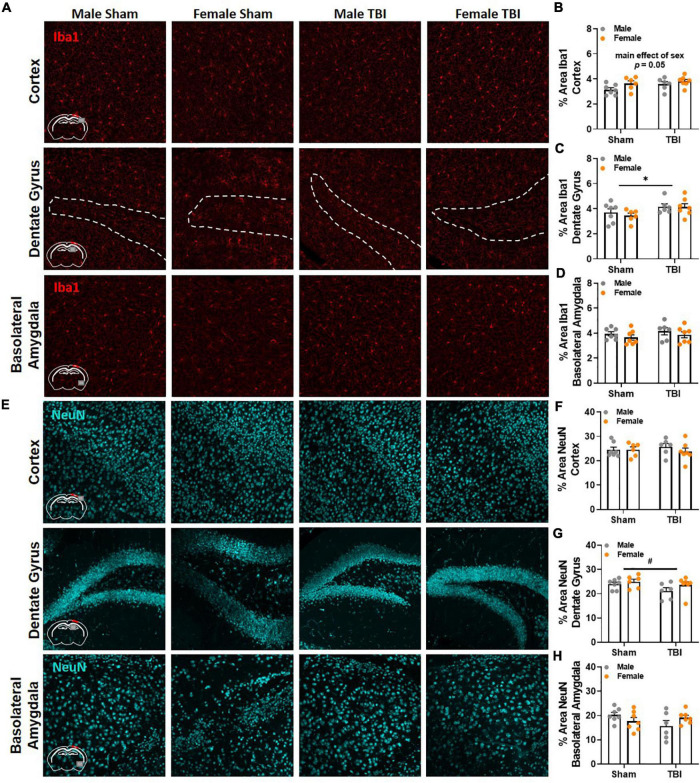
Gross pathological change is absent near the site of injury and in the basolateral amygdala 17 weeks post-TBI. **(A)** Representative images of Iba1 labeling in the ipsilateral primary somatosensory cortex, dentate gyrus, and basolateral amygdala 17 weeks post-injury. **(B)** Percent area of Iba1 was higher in female mice than male mice in the cortex; but a main effect of sex fell short of statistical significance. **(C)** TBI increased percent area of Iba1 labeling in the dentate gyrus. **(D)** No significant differences in Iba1 percent area were detected between experimental groups in the basolateral amygdala. **(E)** Representative images of NeuN labeling in the ipsilateral primary somatosensory cortex, dentate gyrus, and basolateral amygdala 17 weeks post-injury. **(F)** No significant differences in NeuN were detected between experimental groups in the cortex. **(G)** No significant differences in NeuN were detected in the dentate gyrus, but a trending main effect of TBI is present. **(H)** No significant differences in NeuN percent area were detected between experimental groups in the basolateral amygdala. *N* = 6–7/group; error bars indicate SEM. Main effects are identified in the figure. *p < 0.05; ^#^*p* < 0.09.

Finally, brain tissue was collected 17 weeks post-injury to evaluate microglia reactivity and neuronal loss via Iba1 and NeuN, respectively. Brain regions of interest were in close proximity to the epicenter of the injury and included the ipsilateral primary somatosensory cortex, dentate gyrus, and dorsolateral thalamus. We also examined the basolateral amygdala because of its involvement in spatial and conditioned fear memory (Maren, 2003; Pape and Pare, 2010; Wahlstrom et al., 2020). No significant differences in Iba1 and NeuN were identified between experimental groups in the thalamus. Representative images of Iba1 and NeuN labeling in the cortex, dentate gyrus, and basolateral amygdala are included in Figures 6A,E, respectively. A main effect of sex fell short of statistical significance in Iba1 labeling in the cortex, *F*(1,22) = 4.17, *p* = 0.05, Figure 6B. Males (*M* = 3.36, *SEM* = 0.22) had slightly less area covered by Iba1 labeling than females (*M* = 3.73, *SEM* = 0.08). A main effect of injury was identified in Iba1 labeling in the dentate gyrus, *F*(1,22) = 5.03, *p* < 0.05, Figure 6C. No significant group differences in Iba1 percent area were identified in the basolateral amygdala, Figure 6D. No significant differences in NeuN were detected in the cortex, Figure 6F, or basolateral amygdala, Figure 6H; however, a trending main effect of injury was observed in the dentate gyrus, *F*(1,22) = 3.21, *p* < 0.09, Figure 6G.

## Discussion

This study sought to complete a comprehensive analysis of memory impairment after TBI. The experimental design included both pre- and post-injury behavioral assessment. Three cognitive tests were utilized to measure hippocampal-dependent memory (Kennard and Woodruff-Pak, 2011), which is often compromised after TBI. First, the PAL task is hippocampal-dependent and assesses object-location association memory (McTighe et al., 2009; Horner et al., 2013; Kim et al., 2015). Mice acquired the PAL task 22 weeks before sham injury or TBI. Therefore post-injury testing targeted the ability to recall a previously learned task. Second, mice completed the Barnes maze (Barnes et al., 1980), which is a hippocampal-dependent spatial memory task (McNaughton et al., 1986) that also engages other brain regions such as the basolateral amygdala and entorhinal cortex for memory consolidation (Wahlstrom et al., 2020). Third, mice completed fear conditioning which includes both hippocampal-dependent and –independent components and is associated with cholinergic signaling (Gould and Wehner, 1999; Gale et al., 2001; Maren, 2003; Feiro and Gould, 2005; Pape and Pare, 2010). We hypothesized that male and female mice would display unique memory deficits after TBI.

Naïve male and female mice showed no differences in PAL task acquisition, approximately 22 weeks before sham injury or TBI. To date, few experimental TBI studies have incorporated this touchscreen task because of the extensive training required (Nichols et al., 2017; Braeckman et al., 2019). Contrary to previous reports (Nichols et al., 2017), mice performed above chance levels and progressed through training stages over the course of 4 weeks in a standard light dark cycle as outlined by Campden Instruments. Because of concerns regarding retention of the task over several months, mice were retrained in PAL task acquisition procedures 5 weeks before sham injury or TBI. The number of sessions required to reach criteria for punish incorrect and PAL acquisition was reduced by half, which resulted in 3 weeks of retraining. Together, these data show that training for PAL task acquisition is extensive and requires a substantial time commitment. However, both sexes acquire and retain the PAL for up to 20 weeks.

After retraining in the PAL task, all mice returned to free feeding for 2 weeks before sham injury or TBI. This was done to limit the potential influence of food restriction on post-injury recovery. As expected, time to self-right was longer in TBI mice compared to sham injured mice. Sex did not influence the righting response to injury. It is worth mentioning that anesthesia time for surgery and injury as well as the pendulum height on the FPI device were not adjusted for sex and remained consistent between subjects. All mice remained undisturbed with food and water *ad libitum* for 4 weeks post-injury. This was done to ensure that food restriction, which is required for PAL testing, did not confound early recovery after sham injury or TBI. Procedural memory of must touch and punish incorrect remained intact in all mice after injury.

Clinical studies show performance deficits in the CANTAB test following TBI (Salmond et al., 2005; Sterr et al., 2006); however, results from experimental studies are mixed (Nichols et al., 2017; Braeckman et al., 2019). Here, we find that TBI does not impair PAL task performance. Correct choices improved from 50 to 80% over 6 weeks of post-injury testing in all experimental groups. Interestingly, sex differences were detected in session time. On average, males took longer than females to complete a testing session. This likely occurred because latency to make an incorrect choice was longest in male mice. Latency to make an incorrect choice was also lengthened by TBI. Together, these results suggest that male TBI mice may be most vulnerable to delayed choice making over time, particularly when they are incorrect. While these subtle delays in decision making did not influence overall task completion, they may reflect information processing deficits (Folweiler et al., 2018). Indeed, a variety of clinical studies show that information processing speed is compromised after TBI (Hinton-Bayre et al., 1997; Madigan et al., 2000; Johansson et al., 2015).

It is worth acknowledging that all mice were trained on the PAL task several months before injury. Therefore, post-injury assessment evaluated retrograde memory (see review, Paterno et al., 2017). Previous studies show that retrograde memory is impaired after lateral FPI for up to 25 days post-injury (DPI) in rats (Lyeth et al., 1990; Smith et al., 1991, 1994; Hicks et al., 1993). Deficits in retrograde memory are associated with disrupted consolidation, storage, or retrieval of information. However, robust training and a procedural prompt before post-injury testing resolves retrograde memory deficits (Whiting and Hamm, 2008; Sebastian et al., 2013). Therefore, these results are consistent with earlier publications and suggest that experience may compensate for some brain injury induced deficits of remote memories (Sebastian et al., 2013).

Next, we assessed spatial reference memory in the Barnes maze 14 weeks post-injury. This allowed us to evaluate anterograde memory of a recently acquired task, which is most often considered in experimental models of TBI. Anterograde memory deficits occur as a result of disrupted learning or memory. This can include impaired encoding, consolidation, storage or retrieval of information acquired after TBI (Whiting and Hamm, 2008; Whiting and Kokiko-Cochran, 2016). The primary dependent variable of interest was latency to the goal box, which significantly decreased across testing days in all experimental groups. We anticipated that there would be a main effect of injury, and TBI mice would take longer than sham injured mice to reach the goal box. This was not the case though, and actually aligns with another study showing that Barnes maze deficits resolve by 3 months post-injury in rats (Fedor et al., 2010). Interestingly, male TBI mice lagged in latency to reach the goal box on testing days 2–4 compared to all other groups. Because of this, we decided to incorporate additional dependent variables of interest that might better define within and between trial performance.

Mice do not always enter the Barnes maze goal box, which can skew escape latency data. To account for this, other studies have incorporated measurement of primary path length, primary latency, and primary errors. These measurements correspond to the distance traveled, amount of time, and number of errors before the first encounter with the escape hole, respectively (Harrison et al., 2006; Patil et al., 2009). To identify mice that sit near the escape hole without entering, we evaluated time in the goal perimeter. The goal perimeter was operationally defined as a 1.5 inch diameter of space around the escape hole on top of the Barnes maze. We saw that male mice spent the most time in the goal perimeter on testing days 1 and 2, but this behavior resolved thereafter and likely did not contribute to longer escape latencies on testing days 3 and 4. Therefore, we also utilized two learning indices to quantitate within and between day performance (Kokiko-Cochran et al., 2016; Whiting and Kokiko-Cochran, 2016). On average, male mice displayed improved within day performance but impaired between day performance. This response was exaggerated by brain injury. For example, male TBI mice improved by approximately 30 s within any given testing day. However, male TBI mice took almost 15 s longer to reach the goal box between testing days. We hypothesize that this reflects a retrieval deficiency; however, additional studies are needed to isolate this specific component of cognitive processing. Female mice took the shortest amount of time to reach the goal box and therefore a floor effect may prevent a similar trend. Together, these results show that male and female mice display unique within and between day performance. TBI in male mice may exaggerate these differences and contribute to longer testing trials in the Barnes maze 14 weeks post-injury.

Finally, fear conditioning was used to evaluate associative memory 15 weeks post-injury. Deficits in contextual fear conditioning have been reported after lateral FPI for up to 30 DPI (Witgen et al., 2005; Lifshitz et al., 2007; Cole et al., 2010; Smith et al., 2012; Wilson et al., 2017). We expected that TBI would impair both contextual and cued memory of the tone-shock pairing, resulting in heightened freezing behavior. Because of previous reports in rats, we also expected that female mice would display reduced freezing behavior compared to male mice (Maren et al., 1994; Pryce et al., 1999; Gupta et al., 2001). All mice learned to associate the tone with footshock, resulting in increased freezing behavior across the acquisition test trial. No group differences were identified in response to the contextual environment. Intriguingly, male sham mice exhibited the most freezing behavior in the cued response testing trial. Time binned data analysis showed that all experimental groups displayed an increase in freezing behavior during the first 120 s, which did not include any tone. However, subsequent tone exposures elicited more freezing behavior in male sham mice demonstrating a strong memory of the tone-shock association. By comparison, male TBI mice showed reduced freezing behavior with repetitive tone exposure. Female TBI mice froze more than female sham mice at the beginning of the cued retention trial, but this was not as striking and resolved with repetitive tone exposure.

Sex-specific differences in cued fear response have also been detected after repetitive brain injury- with males freezing less than females after TBI (Tucker et al., 2019, 2021). To date, this is the only report showing that lateral FPI results in sex-specific cued fear memory retention deficits. It is worth acknowledging that freezing behavior increased in all experimental groups during the first 2 min of cued testing. This may reflect a generalized increase in fear behavior to the novel environment, which could be prompted by any of the novel stimuli (e.g., scent or inserts that change the visual and tactile experience). Total or percent time freezing can further be confounded by differences in baseline activity. An activity suppression ratio has been used in previous publications to account for this type of inherent variability (Anagnostaras et al., 2000; Lifshitz et al., 2007). The same analysis may be helpful in discriminating within trial fear as well. Interestingly, an animal’s fear response can include freezing or rapid escape from the threat. A recent report shows that female rats display increased “darting” behavior in response to fear conditioning, reflecting an active response to the CS-US association (Gruene et al., 2015). This may also help to explain reduced freezing in female mice, as well as a shift from passive to active response in male TBI mice.

Based on our previous experiments, we expected to see reactive microglia near the site of injury and in the hippocampus and thalamus 17 weeks post-injury (Kokiko-Cochran et al., 2016, 2017). However, we only observed a modest increase in Iba1 percent area in the ipsilateral dentate gyrus following TBI, which was not influenced by sex. Similarly, no statistically significant differences in NeuN percent area were detected in the brain regions of interest; however, a trending reduction in NeuN was present in the dentate gyrus. These data show that TBI does not induce gross pathological changes near the site of injury 17 weeks post-injury; however, other brain regions may be important in modulating behavioral recovery. For example, Barnes maze also engages other brain regions such as the basolateral amygdala and entorhinal cortex for memory consolidation (Wahlstrom et al., 2020). In addition, the hippocampus is required for the US-context association but the amygdala is required for the CS-US association (Kim and Fanselow, 1992; Phillips and LeDoux, 1992; Rudy, 1993; Gerlai, 1998). Therefore, brain regions outside of the hippocampus may be influencing post-injury behavioral change. Unfortunately, we did not detect any group differences in Iba1 or NeuN in the basolateral amygdala 17 weeks post-injury. Because brain tissue was collected a week after the last behavioral test, antibodies such as cFos and FosB would not accurately reflect neuronal populations that were engaged by the behavioral tests included in this project. Future studies may benefit from time dependent tissue collection immediately after a specific behavioral task. Additionally, multi-channel brain recording systems would provide a tremendous amount of information on specific neural substrates involved in post-injury behavior. These experiments are costly and typically require electrode implantation, which could alter behavioral performance and recovery.

This project sought to provide a comprehensive analysis of post-injury cognition, with a focus on memory. To date, few experimental TBI studies include both pre- and post-injury behavioral analysis of retrograde and anterograde memory. We expected TBI to induce striking cognitive deficits in all tests, but this was not the case. Instead, we found subtle and time-dependent differences between male and female mice after TBI. These results highlight the potential influence of experience in preserving components of post-injury cognition. In fact, we cannot rule out the possible benefits of repetitive behavioral testing in this study. For example, this study lasted a total of 40 weeks with just under 15 weeks of behavioral testing. The stimulus of cognitive testing, including learning new tasks after brain injury, may have offered some protection against cognitive impairment in TBI mice. Another goal of this project was to prioritize quantitative analysis of within- and between-trial performance. By incorporating the acquisition and savings index in Barnes maze, as well as binned time analysis of freezing behavior in fear conditioning, we identified unique deficits in male TBI mice compared to other groups. These subtle changes in cognition are clinically relevant and may change over time (Vincent et al., 2014, see Figure 1), thereby emphasizing the value in sophisticated behavioral study design. Together, these data show that retrograde memory of a previously learned task is intact after lateral FPI. However, male mice are more vulnerable to post-injury anterograde memory deficits. Designing studies that include pre- and post-injury behavioral analysis will be effective in identifying sex-specific trends in memory impairment.

## Data Availability Statement

The raw data supporting the conclusions of this article will be made available by the authors, without undue reservation.

## Ethics Statement

The animal study was reviewed and approved by The Ohio State University’s Institutional Animal Care and Use Committee.

## Author Contributions

JF performed all behavioral testing, behavioral data analysis, and wrote the manuscript. SH performed sham and fluid percussion injury, completed brain tissue collection and processing and immunohistochemistry, and imaging analysis and wrote the manuscript. CC and ZZ completed brain tissue collection and processing, immunohistochemistry, and wrote part of the “Methods” Section. KM provided expertise in project design, behavioral testing and TBI, and edited the manuscript. CVH provided expertise in project design, behavioral testing and TBI, statistical analysis, and edited the manuscript. OK-C designed all experiments, provided funding, performed sham and fluid percussion injury, performed behavioral data analysis and interpretation, and wrote the manuscript. All authors contributed to the article and approved the submitted version.

## Conflict of Interest

The authors declare that the research was conducted in the absence of any commercial or financial relationships that could be construed as a potential conflict of interest.

## Publisher’s Note

All claims expressed in this article are solely those of the authors and do not necessarily represent those of their affiliated organizations, or those of the publisher, the editors and the reviewers. Any product that may be evaluated in this article, or claim that may be made by its manufacturer, is not guaranteed or endorsed by the publisher.
